# A formative evaluation of a family-based walking intervention-Furness Families Walk4Life

**DOI:** 10.1186/1471-2458-11-614

**Published:** 2011-08-02

**Authors:** Karen Milton, Paul Kelly, Fiona Bull, Charlie Foster

**Affiliations:** 1 School of Sport, Exercise and Health Sciences, Loughborough University, Epinal Way, Loughborough, Leicestershire, LE11 3TU, UK; 2 British Heart Foundation Health Promotion Research Group, University of Oxford, Old Road Campus, Headington, Oxford, OX3 7LF, UK; 3 School of Population Health, University of Western Australia, 35 Stirling Highway, Crawley WA 6009, Australia

## Abstract

**Background:**

The family unit may be an important mechanism for increasing physical activity levels, yet little is known about what types of family-based interventions are effective. This study involved a formative evaluation of a 12 week intervention to encourage walking as a family based activity. The intervention consisted of several key elements including led walks and tailored resources, as well as remote support provided via the telephone. The project aimed to explore factors associated with successful delivery of the programme and to identify areas of improvement for future implementation.

**Methods:**

A total of nine interviews were undertaken with programme staff who were involved in either the set up or delivery of the intervention. In addition, four interviews and two focus groups were undertaken with participants to explore their experiences of the programme. The analysis involved both deductive and inductive reasoning.

**Results:**

In total, 114 people participated in the programme, which included 36 adults, 10 adolescents and 68 children (≤ 10 years of age). Adult participants reported several barriers to walking including concerns over their children's behaviour and their ability to maintain 'control' of their children. Walking in a group with other families gave parents confidence to go out walking with their children and provided a valuable opportunity for social interaction for parents and children alike. The most successful walks incorporated specific destinations and an activity to undertake upon reaching the destination. Incorporating other activities along the way also helped to keep the children engaged.

**Conclusions:**

The results of this study have highlighted the important contribution that formative research can make in informing and refining a programme to increase appropriateness and effectiveness. The study has helped to highlight the key characteristics associated with delivering a successful walking intervention to young families. It is recommended that practitioners undertake formative research when developing novel health promotion initiatives to help refine the programme protocols.

## Background

Participation in regular physical activity is recognised as one of the most important behaviours associated with the prevention of chronic disease and the promotion of health and well-being. Adults who are physically active have 20-30% reduced risk of premature death, and up to 50% reduced risk of developing major chronic diseases such as coronary heart disease, stroke, diabetes, and some cancers [1]. In children, participation in regular physical activity is associated with the prevention of type II diabetes, maintenance of a healthy weight, and improved skeletal health [2]. The government physical activity guidelines in England state that adults should aim to take at least 30 minutes of at least moderate intensity activity on at least five days a week and that for children and young people a total of at least 60 minutes of at least moderate intensity physical activity each day is needed [1]. Data from the Health Survey for England shows, however, that over 60% of adults and approximately three-quarters of children do not meet these guidelines [3]. Therefore interventions to promote physical activity among both adults and children are needed.

The 'family unit' has been identified as an important mechanism for increasing levels of physical activity [4,5]. Parental support and sibling participation in physical activity have been shown to predict childhood physical activity [5]. In addition, child care commitments are a frequently cited barrier to physical activity participation [6]. Therefore engaging in physical activity as a family unit is one approach to reducing this barrier. However, very little research has been undertaken to determine what type of family-based interventions are effective at increasing physical activity levels.

In 2009, the Department of Health in England commissioned the Ramblers; Britain's biggest charity working to promote walking, to develop and pilot a family based walking intervention. Walking was the selected activity for the intervention for several reasons; it is free of charge, does not require specialist equipment or facilities, and can be easily incorporated into everyday life. In addition, walking is an ideal introduction to physical activity, especially for people who are overweight or extremely unfit [7], and is viewed as a particularly acceptable and accessible form of activity among populations with low physical activity prevalence [8,9].

The Ramblers intervention, entitled 'Furness Families Walk4Life', was a community based programme to promote walking in a family group, for leisure, exploration and on everyday journeys. Consistent with review level evidence on effective walking interventions, the Walk4Life programme consisted of several key elements including led walks and tailored resources, as well as remote support provided via the telephone [10]. The pilot project was delivered in partnership with Action for Children, a charity which supports children and families through the provision of family-oriented interventions such as baby and toddler groups and parenting classes.

Barrow-in-Furness, a town of 72,000 inhabitants in North West England, was selected to pilot the programme. Barrow-in-Furness is one of the Department of Health's 'spearhead' Primary Care Trust (PCT) areas, meaning it has some of the highest instances of health inequalities and deprivation in England. Action for Children has eight children's centres across Barrow-in-Furness, five of which were selected to deliver the Walk4Life programme.

This paper reports results from the evaluation of the Walk4Life intervention. Emphasis was placed on 'formative evaluation' due to its importance when developing innovative approaches to health promotion [11]. The key evaluation questions were: to explore the barriers to walking among families with young children; to determine whether partnership working with existing service providers is an effective approach to promoting walking; to determine the overall appropriateness of the programme; and to identify areas of improvement for future implementation.

## Methods

### The intervention

The intervention was predominantly aimed at families with children aged two to 11 years; however families with younger or older children were also welcome to take part. The project involved four key components; (1) a four week period of led walks, (2) a resource pack, (3) a seven week period of independent walking and (4) a celebration event at week 12 to mark the end of the programme. The programme was advertised via leaflets and posters in Action for Children's centres across Barrow-in-Furness and 1,500 leaflets were posted to households within the local area. The project was advertised in local venues such as cafes and schools and also featured on local radio and in several local newspapers.

The four week led walk programmes were organised and delivered by a Walk4Life Project Officer, with assistance from Action for Children staff who also attended the walks ('support walkers'). The Project Officer had a background in childcare and was employed on a fixed-term contract for the duration of the pilot project. The led walks ran from different centres on different days (Monday to Friday) and at a variety of time-slots between the hours of 09:00 and 16:00. The days and times of the walks were determined by the Project Officer in consultation with Action for Children staff. Week one involved an informal workshop focused on the benefits of regular walking as well as the barriers to walking and how to overcome them, which was followed by a short walk. Each programme involved three further weekly walks which started and finished at the same centre. Each walk took a different route and was designed to be safe and easy for children, while incorporating places of interest, including areas of green space as well as everyday destinations such as shops and transport interchanges. Each walk was designed to last for approximately 40 minutes and was themed around one or more simple messages - walking is healthy, walking is fun, walking is green, walking for adventure, walking with friends and family, and walking safely. Refreshments were provided at the children's centre at the end of each walk. One 'Trail Tales' resource pack was provided to each child, and contained a log book and stickers for children to record the amount of walking that they undertake and a set of story books tailored to match the needs and interests of the child(ren). Participants were given instructions for the use of the resource pack by the Walk4Life Project Officer. This included an introduction to the various activities included within the pack, as well as how to use the log book to record the amount of walking undertaken. Support was also provided at the centres before and after the walks.

Following the four-week period of led walks it was intended that families would continue to meet at the centre and undertake group walks independently, without the leadership of the Project Officer. The Project Officer made phone calls to each of the families at week five and week seven as a means of maintaining contact and providing encouragement and support. All families were contacted regardless of how many times they had engaged with the programme. The phone calls were used to encourage families to use the resource pack to gain ideas and inspiration about different types of walks and activities to undertake within the local neighbourhood. A letter was sent to participants' home addresses inviting them to attend a celebration event at week 12 to mark the end of the programme and encourage the families to continue walking. The celebration event included a walk and games as well as a presentation with certificates and prizes for the individuals who took part in the greatest number of walking events throughout the programme.

### Evaluation

An independent research team were appointed to undertake a formative evaluation of the Furness Families Walk4Life project. The primary aims of the evaluation were to determine the barriers and facilitators to implementation and to identify the key factors associated with successful delivery of the programme. Formative evaluation was considered appropriate given the project's early stage of development [11,12]. Qualitative data collection methods were used as they are exploratory in nature and therefore useful for gaining insight into the appropriateness of the pilot intervention. A total of nine interviews were undertaken with programme staff who were involved in either the set up or delivery of the intervention. This included staff from both the Ramblers and Action for Children with a range of roles and seniority. In addition, a coordinator from another local walking intervention agreed to be interviewed. A semi-structured interview guide was developed by the evaluation team, and themes included the initial set up of the programme, experiences of recruiting families, delivery of the 12 week intervention, and recommendations for future practice.

In addition, four interviews and two focus groups were undertaken with participants to explore their experiences of the programme. All adult participants in the programme (aged 16+ years) were invited to contribute to the qualitative evaluation. In total, 11 participants agreed to attend an interview or focus group (male = 2, female = 9). Participants had attended between two and four walks during the led walk phase of the intervention. A semi-structured questioning route was developed and themes included motivation to take part, experiences of the led walks, usefulness of the resources, ability to maintain walking levels during the independent walking phase of the programme, and recommendations for future implementation. All interviews and focus groups were conducted by a member of the evaluation team [PK and KM] and took place at a variety of the Action for Children centres which were involved in the delivery of the programme. Interview duration was 25-40 minutes and focus groups lasted for 60 minutes. All interviews and focus groups were recorded on a digital audio device with consent.

This evaluation was classified by the National Research Ethics Service as an 'audit' of services as opposed to 'research' and consequently full ethical approval was not required. Informed consent, however, was obtained from all participants who took part in the evaluation, which included agreement to use direct quotes in any reports on the findings.

### Data analysis

The interview and focus group recordings were transcribed verbatim and independently analysed by two researchers [KM and PK]. Although the interview schedules were semi-structured around key themes of interest such as 'experiences of the led walks', the questions were open-ended, allowing exploration of the issues which were important to the participants, as opposed to being pre-determined by the researchers. The analysis therefore involved both deductive and inductive reasoning [13]; deductive to group the data based on each theme within the interview schedule (e.g. experiences of the led walks) and inductive to identify the key issues identified within each theme (e.g. social interaction, appropriate destinations, and duration of the walks). Data were managed and organised using N-Vivo qualitative software package.

## Results

Ten programmes were delivered across the five Action for Children centres between May and August 2009. In total, 114 people participated in the programmes, which included 36 adults, 10 adolescents and 68 children (≤ 10 years of age). Adult participants were typically female (9 male), aged between 16 and 44 years, and all were classified as White British. The programme was predominantly aimed at families; however the led walks attracted a local nursery group and also a teen group, which explains the high proportion of children to adults. Although quantitative data on baseline walking levels were not available, adult participants reported that they rarely went walking as a family due to concerns over their children's behaviour and their ability to maintain 'control' of their children while out walking.

In total, 11 participants took part in the qualitative evaluation, which reflects approximately one third of adult participants in the programme. In addition three members of staff from the Ramblers, six staff from Action for Children, and a representative from an existing walking intervention in the local area were all interviewed. The results of the interviews with programme staff and the local walking coordinator are presented, followed by findings from the interviews and focus groups with programme participants.

### Programme staff and stakeholders

A total of nine interviews were undertaken with programme staff who were directly involved in either the set up or delivery of the intervention. In addition an interview was conducted with a walking coordinator for an existing intervention in the local area. The results are presented in the following theme areas: working in partnership; planning and preparation; and programme delivery.

#### Working in partnership

The partnership between the Ramblers and Action for Children was initially established on a strategic level, with mutual support for the development of the programme from high level staff at both the Ramblers and Action for Children. The project was seen as an attractive way for both organisations to achieve common or shared outcomes, namely delivering healthful development activities and promoting family time. The Ramblers brought experience and expertise in the promotion of walking and Action for Children facilitated access to the target group. The value of this partnership was emphasised by staff at both the Ramblers and Action for Children;

"[The Ramblers] have got a good coverage of volunteers across the country and walking across the country but not really the expertise or contacts to do it with children or families. So we needed help in that direction and Action for Children have got that national spread of working with children and contact through family centres throughout the country"

(The Ramblers Walk4Life Project Officer)

"It fits with our values, it fits with our mission, it fits with what we are trying to do and it also adds value to our services, because it is another partner coming in and helping us to deliver the targets that we have"

(Action for Children Strategic Development Manager)

Once the partnership was established at a strategic level the Ramblers appointed a Walk4Life Project Officer to facilitate the development of partnerships at a local level. The Project Officer reported that it was challenging to get the programme 'off the ground' while initially working remotely, however once contact and introductions were made with the children's centre staff, project organisation at a local level was more collaborative, and this was felt to have aided local "buy-in and engagement".

In terms of wider relationships, the pilot successfully engaged other community initiatives and personnel despite initial feelings of animosity. Our interviews indicated that existing projects can be territorial and resent new initiatives in their field. In an interview with a walking coordinator for an existing initiative in the area, it became clear that the project benefited from efforts to communicate their goals and desire to work alongside, rather than replace, existing initiatives;

"...I was horrified when I heard about the project because it seemed to cut right across what I was supposed to be doing, but then I thought for 10 minutes and I thought 'I haven't got into those places', it is an area I don't touch because my typical client is a 65 year old lady with a bad knee or a heart problem. It was very welcome in the end and they did an amazing amount with the time they had"

(Local walking coordinator)

#### Planning and preparation

Due to the Department of Health's timelines for the project, the Ramblers had approximately four weeks from learning that they were the successful applicant to deliver the programme to the date that the first intervention was due to take place. Within this time period the Ramblers had to establish links with Action for Children at a local level, select appropriate centres from which to run the pilot, plan the walking routes from each of the five centres, advertise the programme, and appoint a Project Officer to run the programme. The Head of Walking Programmes and Promotion from the Ramblers highlighted the challenge presented by this short lead-in time in delivering a successful programme on-the-ground. This was echoed by Action for Children staff;

"In that time we had a good relationship with Action for Children at a strategic level, but that is very different to actually what is needed to make a pilot run 300 miles away in Barrow"

(The Ramblers Head of Walking Programmes and Promotion)

"Everybody was really optimistic but I think there wasn't enough planning before the first walk. I think the promotion time before it started wasn't enough and it was all very quick"

(Action for Children employee in Barrow-in-Furness)

#### Programme delivery

Action for Children recognised the importance of employing the Project Officer (Ramblers) who was on-the-ground in Barrow-in-Furness and able to form and maintain relationships with centre staff during the delivery of the programme. A similar programme organised by another agency had failed, due to insufficient support being provided to the Action for Children centre staff;

"Because we are under pressure to deliver services, our staff are always working to full capacity...the Ramblers' decision to actually employ somebody was something I was fairly keen to happen...we had another pilot running alongside this which did not become so well established, and that was because we were expected to run it ourselves and we didn't have the capacity"

(Action for Children Strategic Development Manager)

The Project Officer reported that making links with the centre administrative staff was crucial to delivery. As the 'front of house' staff they had daily contact with and knowledge of centre users and the wider community;

"I got to know all of the admin and front desk reception staff. They are the first port of call, they are the ones that book the rooms, they know what's going on, they know the programme schedule and if you can fit in. They know when people are coming in and out and know what's going on in that centre. They're your hub of information and so I always made sure I knew all of them"

(The Ramblers Walk4Life Project Officer)

The 'support walkers', who were Action for Children staff who attended the walks, were difficult to recruit. Those that were successfully engaged were considered influential in the delivery of the programme and consistency in personnel made a difference to group bonding and adherence to the programme. Feedback from centre staff suggests that support walkers were challenging to recruit due to a lack of communication. It may be important to engage volunteer support walkers during the development phase of the programme which was not the case in Barrow-in-Furness. Those that were recruited reported they were attracted not by the health aspects but by the chance to combine the walks with social interaction for the centre users;

"When the Health Authority is pushing something and they are pushing to become healthy, it is pushed so hard that you need to do this, this was just a different type of concept... It wasn't so much exercise as people getting together socially, which is different to the way most health options are promoted"

(Action for Children employee in Barrow-in-Furness)

### Programme participants

In total, eleven programme participants contributed to the evaluation. The results are presented in eight thematic areas: partnership working with existing service providers; marketing; motivation to take part in the programme; experiences of the led walks; benefits of taking part in the programme; impact of the programme on attitudes and walking behaviour; usefulness of the resources; and recommendations for future implementation of the programme. Where direct quotes are provided, details have been given of the gender of the participant, the number of children that they have and whether they took part in an interview or focus group.

#### Partnership working with existing service providers

Utilising an existing and well recognised family-oriented service provider to deliver the programme was well received and helped to encourage the families that the programme was appropriate for them. In addition, the centres were viewed as a good meeting place for the walks as the target group are aware of the centres and they are centrally positioned and easily accessible;

"There's not really anywhere else you could meet up. I don't think there's anywhere else like that. It's not far from anywhere really is it? School is just up the road and people live round here, buses run down here, there's a bus stop right outside"

(Participant 1; female teenager (aged 16) who took part in the walks; no children; interview 1)

#### Marketing

Although the programme was advertised via a number of avenues including newspapers and local radio, the majority of participants who took part in the programme were not aware of this advertising and found out about the walks via a leaflet in the Action for Children centres. The programme therefore predominantly attracted families who were existing users of the centres. Advertising in the jobcentre, sports centre and doctors' surgeries were recommended for future marketing campaigns. Also, in relation to future marketing of the programme, it was suggested that word-of-mouth may be the most effective strategy.

The way in which the scheme was marketed as a social opportunity as opposed to pushing the health agenda was viewed positively and was perceived to be useful in attracting people to the programme;

"...it was families getting together...mothers can have a chat and we can all go walking together...it was nice just to get out of the house"

(Participant 7; mother of two; focus group 1)

"Well it is something to do with children, isn't it, and company-somebody to do it with...if you have got two little ones it is hard work walking on your own"

(Participant 5; mother of two; focus group 1)

#### Motivation to take part in the programme

Families with young children were generally looking for activities to amuse the children at times when they were not at nursery. They reported a lack of opportunities, particularly in the afternoons. The programme was attractive to young families as it was free of charge and was also viewed as a good opportunity to spend time as a family;

"It is more interesting because you are with them, do you know what I mean? You are doing something with them, whereas you go up to the Playdome and you pay £7 and [parents] just sit there"

(Participant 5; mother of two; focus group 1)

#### Experiences of the led walks

Participants enjoyed taking part in the programme and described the walks as "fun". By far, the most enjoyable aspect of the programme reported by the participants was social interaction with other families;

"I can take him for a walk any time I want by myself, but I think the interaction with other kids and other adults is quite important"

(Participant 2; father of one; interview 2)

Parents emphasised the importance of having a destination to reach as part of the walks, as this was viewed as a "goal" by the children. The walk leader often provided a list of things to look out for, such as wildlife, zebra crossings and local landmarks, and this was useful for helping to engage the children. Incorporating activities into the walks, for example kite flying or feeding the ducks was also viewed positively;

"They went to fly kites and they absolutely loved it, they really did"

(Participant 8; mother of three; focus group 2)

Participants reported discovering areas of Barrow-in-Furness which they never knew existed. In addition, participants tended to enjoy the walks in parks and green spaces but some parents did not like the walks which took place around the town centre;

"It was quite nice because we were going through nice areas, places that I never even knew existed. There was a pond and that and it was like "how long has that been there?" because I had no idea"

(Participant 7; mother of two; focus group 1)

"Just one (walk) I didn't think was very good was the town walk. There was just the two of us with the four boys and it was just quite stressful really, because you have busy roads and they are lively boys, and you were having to try and keep them out of the shops and really I don't think there was anything for the children on that walk...The other walks you went to feed the ducks or you went to a park, but that one it was through town and back to the centre"

(Participant 6; mother of two; focus group 1)

Participants reported that 40 minutes was an appropriate duration for the walks. It was also suggested that there could be 'escape routes' should, for any reason, the families want to return to the Action for Children centre before the end of the walk.

#### Benefits of taking part in the programme

By far, the most important aspect of the programme to the participants was social interaction with other families. The social aspect of the programme, and having other adults around, made parents feel more confident about being out walking with their children;

"If you have got two little ones it is hard work walking on your own, because one goes one way and the other goes the other way, so it was nice to do it in a group"

(Participant 5; mother of two; focus group 1)

For one participant who attended a group with low participation rates, the lack of social interaction on the walks was disappointing;

"The best part of it I think, although it was limited in this group, is the whole group interaction, other adults and other children, and that is the best part of it, I think. He [participant's son] is not just going out and getting bored walking with me, he has got other children to play with and other adults to talk to and stuff. So that is the best part of it. But as I say, unfortunately that was a bit limited in this group"

(Participant 2; father of one; interview 2)

In addition to the social benefits of the programme, participants reported "feeling better" being out in the open, developing confidence, and weight loss.

#### Impact of the programme on attitudes and walking behaviour

Participants reported that the programme had made them "feel a bit better about walking" and made them "enjoy walking more". Taking part in the programme also raised participants' awareness of the amount of walking that they do. The majority of participants had attempted to continue walking after the four week period of led walks, although they tended to walk as a family as opposed to meeting up with other families at the centre. It was felt that four weeks may be insufficient to establish the social cohesion for the families to continue walking as a group. For some families, a change in the parents' attitudes towards walking resulted in more walks being undertaken, but in some cases the walks were instigated by the children;

"She (my daughter) sits there and starts getting bored, because she doesn't like being in. "Do you want go for a walk?", "yes, come on". And yesterday even though I was ill, "all right, come along, we will have a walk"

(Participant 7; mother of two; focus group 1)

#### Usefulness of the resources

Each family received a resource pack containing an activity log, stickers and a series of story books and views were mixed. Some families completed the log of activities and enjoyed keeping a record of the walks that they had undertaken. Others viewed this as "homework" and reported feeling "pressure" from the Project Officer to complete the activity logs;

"She has got a book at home now with all the flowers and stuff that she picked on the walks... so we bought her one, it was a photo album but now it has just got little, in the little sleeves, it has got little flowers and stuff and that is her remembrance from her walks. Even now if we go out and she finds a flower she likes, she will pick it and it will go in my bag or the front of the pram and then when we get home it has to come out and it has got to go in to her book. We are going to have a book full of dead weeds"

(Participant 7; mother of two; focus group 1)

"We come for enjoyment, you know what I mean, you don't want to go home and start doing homework"

(Participant 5; mother of two; focus group 1)

In terms of improvements to the resource pack it was suggested that it could contain more walking routes to facilitate independent walking, and also extended routes to be attempted over time.

#### Recommendations for future implementation of the programme

The walks ran from different centres on different days and at a variety of different time slots, which helped to fit with the differing schedules of the participating families. Two participants suggested improvements for the scheduling of the walks. Firstly, the early morning walks were not suitable for parents with children old enough to attend school because there was insufficient time to get from the school to the Action for Children centre. Scheduling this walk at a slightly later time would allow more families to attend. Secondly, it was suggested that a walk that took place after 4 pm would allow families with older children to attend after school. In addition, some families did not attend the walks on days when it was raining. It was suggested that Action for Children could organise alternative indoor activities for days on which the weather was not conducive to walking.

## Discussion

This study focused on undertaking a formative evaluation of a family-based walking intervention to determine the appropriateness of the intervention and to refine the programme for dissemination on a larger scale. Key lessons were learned from both programme staff and participants about how to deliver family-based interventions to promote physical activity and specifically walking.

Walking was viewed as a particularly acceptable form of physical activity among the participants as it is free of charge and also facilitated family-time. Many physical activity interventions are targeted at either adults or children and do not allow for interaction as a family unit. This is a particular strength of the intervention.

It emerged that some adult participants, particularly single-mums, do not walk anywhere with their children due to concerns about behaviour and maintaining 'control' of the children. Walking in a group with other families gave parents confidence that they had support around them if they needed help. Also the children had other children to interact with on the walks which helped to maintain their interest. Participants emphasised the importance of social interaction as a facilitator to participate in the programme and as a motivating factor to continue to take part. This is supported by previous research which reports that social networks have an important influence on changing behaviours such as increasing physical activity [14]. Therefore a key aspect of future interventions to increase physical activity should be on building, strengthening and maintaining social support. Guidance documents such as the Partnership for Prevention Action Guide [14] should be consulted for ideas on how to maximise these aspects of programme delivery.

Several characteristics of the walks were viewed as important and should be taken into consideration when developing walking interventions for families and/or children. The most important feature was fun and adventure. Having a particular destination to reach on the walk was important for providing a focus, and activities to undertake along the way also helped to keep the children engaged. The most successful walks were those which incorporated an activity upon reaching the destination, for example kite flying or feeding the ducks. Walks in areas of green space were preferable to walks in town as they were free from the stresses of busy roads and also allowed the children more freedom to play.

The programme ran on weekdays only. Interestingly an additional walk was scheduled to take place on a Saturday afternoon but was cancelled due to no attendance for the first two weeks. This suggests that weekday walks may be preferable for the target group over walks which take place on a weekend. Although the timing of the walks suited the young families, some parents and particularly those with older children, expressed a need for greater flexibility in terms of the timing of the walks to enable families with older children to attend after school. Parents also expressed the need for greater options in terms of the distance of the walks. This aspect of programme delivery is crucial when working with young families as a specified target group due to the wide range of fitness levels and abilities of both the parents and children.

Although the four week led walk programme provided an opportunity for the families to interact, this time-frame was insufficient for families to establish group cohesion and continue to meet and walk as a group. A longer period of structured led walks is likely to facilitate this process. Although the families did not meet as a group, they did continue to walk independently and found the resource pack useful for developing ideas on how to make the walks fun for the children. It is important, however, to consider the usefulness of the resource beyond the 12 week intervention period. To ensure longevity of the resource requires variety and diversity in terms of the walks and activities provided as well as progression to enable participants to attempt longer walks over time.

In the current project the delivery partnership was a particular strength as both organisations had similar visions and objectives while being able to contribute complementary elements to the programme. The Ramblers have extensive experience of planning routes and delivering walking interventions. Action for Children are experienced in delivering family-oriented services and helped to facilitate access to the target group. In addition, the Action for Children centres were conveniently located and an ideal meeting place for the walks. These findings are supported by previous research which has highlighted the importance of a trusted or familiar agency for participant recruitment [15]. A challenge in terms of sustainability of the programme is capacity and resources. Action for Children staff expressed that they would not be able to deliver the programme without the full-time Ramblers employee who coordinated and delivered the programme. Further research is warranted to understand how these types of interventions may become embedded into existing family-oriented services, without the need for additional bought-in staff. Engaging volunteers may be one potential solution.

An important consideration for future implementation of the programme is marketing. In the current study, participants mainly found out about the programme through the Action for Children centres and were not aware of the wider advertising campaign. It is possible that the programme was not advertised using appropriate channels, that the content of the advertisements where not effective at encouraging participation, or that the short lead-in time did not allow sufficient exposure to the various marketing strategies. Word-of-mouth was recommended by the participants for future promotion of the programme, which is also supported by previous research [16]. A longer lead-in time and a longer intervention period would facilitate this recruitment strategy.

In summary, key characteristics of a walking intervention aimed at young families include: delivery of the programme in collaboration with established and respected providers of family-oriented services, flexibility in scheduling and avoiding conflicts with school hours; incorporating areas of green space; an end destination to reach on the walk; and an activity to undertake upon reaching the destination. The walks should also incorporate activities along the way, for example looking for a pre-set list of items, including street signs, wildlife or local landmarks. Walks should last for approximately 40 minutes, although planning 'escape-routes' into the walks will help to ensure they are appropriate for a range of age groups and abilities. Finally, every effort should be made to promote social interaction as a means of engaging young families in the walks and to facilitate an enjoyable experience whilst taking part.

A strength of the evaluation is that in the focus groups and interviews we were able to speak to participants and staff at all levels of seniority, from strategic development to those employed in on-the-ground implementation. The evaluation reflected the experiences of those involved in the project from conception through to completion. The open-ended nature of the interview schedules allowed the participants to describe the issues which were important to them. Formative evaluation is often overlooked during the development of interventions and yet these results highlight the important contribution that this phase of research can make to informing and refining a programme to increase appropriateness and effectiveness. Future health promotion interventions should be encouraged to undertake formative research during this development phase.

Several limitations should be noted. Men were under-represented in the programme. Previous research suggests that women are more likely than men to use children's centres, which may explain the higher proportion of women recruited to the study [17]. This may reflect the respective roles of parents in terms of childcare, rather than children's centres and/or family oriented interventions being inappropriate for men. Still, further research is needed to determine how to attract more male participants to these types of interventions. All participants in the programme were White British. This was not unexpected, as 96% of Barrow-in-Furness' population are White British, with just 4% ethnic minority populations [18]. However, it is unclear to what extent these results will be generalisable to other population or ethnic groups. The programme participants who took part in this evaluation were self-selected. It is possible that the experiences of these participants differ from those who were not willing to take part. In addition, we did not interview anyone who had tried the programme once and never returned, nor people who had heard about the programme but decided not to take part.

## Conclusions

This formative evaluation was undertaken to determine the appropriateness of an intervention for encouraging walking as a family-based activity. Several key aspects of the programme were associated with successful delivery, including partnership working with existing family-oriented service providers, a focus on social interaction for both parents and children, and key characteristics of the walks including a destination. It is recommended that practitioners consider these aspects of programme implementation when delivering walking interventions to young families. It is also recommended that practitioners undertake formative evaluation to determine the appropriateness of new initiatives to promote physical activity, to ensure the intervention is appropriate for the target group and to inform refinements to the intervention protocols.

## Competing interests

The authors declare that they have no competing interests.

## Authors' contributions

All authors developed the study protocols. PK and KM undertook the interviews and focus groups, analysed the data and prepared a first draft of the manuscript. CF and FB assisted in revising the manuscript. All authors read and approved the final manuscript.

## Funding Source

The Furness Families Walk4Life project, including the evaluation, was funded by the Department of Health.

## Pre-publication history

The pre-publication history for this paper can be accessed here:

http://www.biomedcentral.com/1471-2458/11/614/prepub
